# Atomic Layer Grown Zinc–Tin Oxide as an Alternative Buffer Layer for Cu_2_ZnSnS_4_-Based Thin Film Solar Cells: Influence of Absorber Surface Treatment on Buffer Layer Growth

**DOI:** 10.1021/acsaem.2c02579

**Published:** 2022-10-14

**Authors:** Natalia M. Martin, Tobias Törndahl, Melike Babucci, Fredrik Larsson, Konstantin Simonov, Dorotea Gajdek, Lindsay R. Merte, Håkan Rensmo, Charlotte Platzer-Björkman

**Affiliations:** † Solar Cell Technology, Department of Materials Science and Engineering, Uppsala University, SE-751 21 Uppsala, Sweden; ‡ EVOLAR AB, Uppsala 756 51, Sweden; § Molecular and Condensed Matter, Department of Physics and Astronomy, Uppsala University, SE-751 21 Uppsala, Sweden; ∥ Department of Materials and Process Development, Swerim AB, P.O. Box 7047, SE-164 07 Kista, Sweden; ⊥ Department of Materials Science and Applied Mathematics, Malmö University, SE-211 19 Malmö, Sweden

**Keywords:** kesterite CZTS, ALD ZTO, interface characterization, N_2_ annealing, XPS, HAXPES, XAS

## Abstract

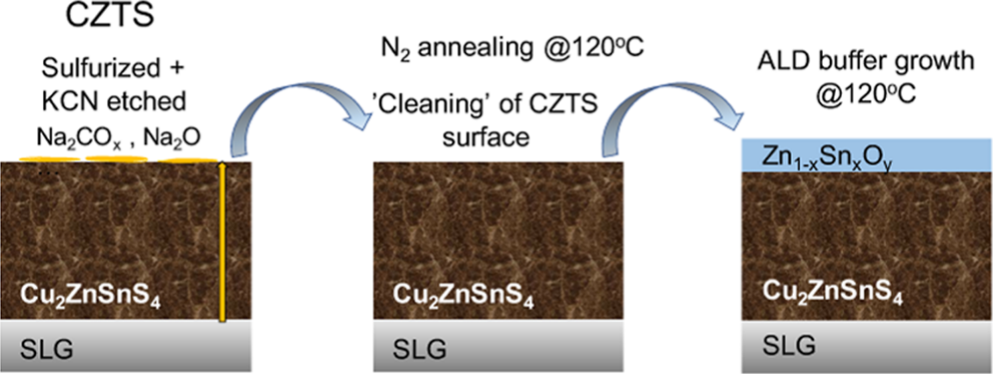

Zn_1–*x*_Sn_*x*_O_*y*_ (ZTO) deposited by atomic layer deposition has shown promising results as a buffer layer material for kesterite Cu_2_ZnSnS_4_ (CZTS) thin film solar cells. Increased performance was observed when a ZTO buffer layer was used as compared to the traditional CdS buffer, and the performance was further increased after an air annealing treatment of the absorber. In this work, we study how CZTS absorber surface treatments may influence the chemical and electronic properties at the ZTO/CZTS interface and the reactions that may occur at the absorber surface prior to atomic layer deposition of the buffer layer. For this, we have used a combination of microscopy and synchrotron-based spectroscopies with variable information depths (X-ray photoelectron spectroscopy, high-energy X-ray photoelectron spectroscopy, and X-ray absorption spectroscopy), allowing for an in-depth analysis of the CZTS near-surface regions and bulk material properties. No significant ZTO buffer thickness variation is observed for the differently treated CZTS absorbers, and no differences are observed when comparing the bulk properties of the samples. However, the formation of SnO_*x*_ and compositional changes observed toward the CZTS surface upon an air annealing treatment may be linked to the modified buffer layer growth. Further, the results indicate that the initial N_2_ annealing step integrated in the buffer layer growth by atomic layer deposition, which removes Na–CO_*x*_ species from the CZTS surface, may be useful for the ZTO/CZTS device performance.

## Introduction

Kesterite Cu_2_ZnSnS_4_ (CZTS) provides an attractive low-cost, earth-abundant, non-toxic absorber layer material for thin film solar cells.^1^ Nevertheless, the record lab efficiency of CZTS-based thin film solar cells is limited to ∼13%,^2^ and higher efficiency is required for industrial implementation, motivating research in this direction. To further improve the device performance, a detailed understanding of the absorber layer surface including its chemical and electronic structure is needed. However, the absorber surface structure can change during the subsequent annealing steps in the device stack formation. A surface structure and composition change driven by the environment may induce strong changes in the solar cells’ properties, and increased solar cell performance has already been reported for some annealing treatments of both sulfide- and selenide CZTS(Se)-based thin film solar cells.^3−7^ In particular, in our recent publication, we showed that a surface treatment of the CZTS absorber prior to the CdS buffer layer deposition results in increased device performance, which was linked to the formation of interface species at the CdS/CZTS heterojunction.^8^ Further, the results show increased device performance upon a surface treatment of the absorber (air exposure or air annealing) as compared to a non-treated surface.

Even though high-performance kesterite devices have been fabricated using CdS as the buffer layer, the use of alternative Cd-free buffer layers is under intensive research in the view of device performance improvements and toxicity issues. Recent work in our lab showed that Zn_1–*x*_Sn_*x*_O_*y*_ (zinc–tin-oxide, ZTO) by atomic layer deposition (ALD) resulted in better open circuit voltage values and a high-quality interface both in terms of band alignment and interface formation.^10^ The highest efficiency using ALD ZTO buffers for CZTS is around 10%, and further optimization of the ALD process is needed.^11−13^

It is seen by us and reported by many other groups that soft annealing of the CZTS film in different atmospheres prior to ALD can change and sometimes improve the device performance drastically.^3,14^ For example, it has been shown that heating up of the CZTS absorber in the ALD reactor, prior to buffer layer growth, has a positive impact on the open circuit voltage, in addition to the positive impact from the ZTO layer itself.^14^ Still, the CZTS surface chemistry during such annealing treatments and its relation to the ALD layer growth are not fully understood, even though changed ZTO material properties could influence the solar cell device performance. Further, the thin film solar cell samples investigated here are polycrystalline, with lateral and vertical inhomogeneities, which may also influence the device performance.

In this work, the goal is to answer some of the open questions in applying ZTO as a buffer layer regarding interface formation with the CZTS absorber such as how the absorber surface treatment influences the properties at the buffer/absorber interface and how reactions occurring at the absorber surface influence the ALD buffer layer growth. For this, we have used a combination of synchrotron-based photoelectron spectroscopies with variable information depths [X-ray photoelectron spectroscopy (XPS), high-energy X-ray photoelectron spectroscopy (HAXPES), and X-ray absorption spectroscopy (XAS)], allowing us to perform an in-depth analysis of the CZTS near-surface and bulk chemical and electronic properties. In particular, HAXPES has been used to study the chemical and electronic properties of the ALD ZTO/CZTS interface for differently surface-treated CZTS absorbers. Complementary XAS and cross-sectional transmission electron microscopy (TEM) measurements give further insights into the bulk properties of the CZTS absorbers exposed to different surface treatments. Further, depth varied photoemission spectroscopy techniques (XPS and HAXPES) were employed to probe how the CZTS near-surface properties change under a mild N_2_ annealing treatment that mimics the annealing treatment typically employed before the ALD buffer layer deposition takes place. The structural/chemical characterization has been complemented by the electro-optical characterization of solar cell devices by current–voltage and quantum efficiency measurements.

## Experimental Section

### Sample Preparation and Device Characterization

The CZTS absorbers were prepared by sputtering from CuS, ZnS, and SnS targets on a Mo-coated soda lime glass (SLG) substrate and later sulfurized in a S atmosphere as we previously described.^8^ The bulk composition of the absorber, as determined by X-ray fluorescence (XRF), was very similar to the sample composition as presented in our recent study [Cu/Sn = 1.91 and Zn/(Cu + Sn) = 0.37].^8^ For the surface treatment studies, the CZTS absorbers were either exposed to air (AE) for 24 h under ambient conditions in a clean room environment or air annealed (AA) on a hot plate (80 s at 300 °C, followed by 10 min at 200 °C). The samples were further etched in 5% potassium cyanide (KCN) (2 min in 1.5 M aqueous solution at room temperature, followed by a H_2_O rinse) prior to buffer layer deposition or XPS analysis. The Zn_1–*x*_Sn_*x*_O_*y*_ buffer layer films were deposited by ALD in an F-120 Microchemistry reactor at 120 °C as previously described.^15^ Similar to previous studies in our lab, the CZTS substrates were loaded into the ALD chamber 30 min prior to ZTO film deposition for temperature stabilization. A total of 700 cycles (or 1000 cycles for the ZTO reference sample) were deposited using a 1:1 ZnO/SnO_2_ super cycle approach with pulse lengths of 0.4:0.8:0.4:0.8 s for DEZn/TDMASn/N_2_/H_2_O/N_2_, respectively, yielding a XRF composition of Zn/(Zn + Sn) = 0.82 for 700 cycles and a thickness of *t* = 34 nm on SLG [or Zn/(Zn + Sn) = 0.79 for 1000 cycles and a thickness of *t* = 47 nm on SLG].

The as-prepared absorber (KCN etched) and buffer/absorber samples described above were sealed in a plastic bag under N_2_ and transported to the synchrotron for characterization. At the beamline, the samples were briefly exposed to air while being mounted on the sample holder and then transferred into the UHV system.

One half of each sample was later processed into devices by sputter deposition of an i-ZnO/ZnO:Al bilayer and mechanical scribing to define cells with an area of 0.05 cm^2^. Dark and illuminated current–voltage (*I*–*V*) measurements were performed using a Newport IV ABA solar simulator. External quantum efficiency measurements to determine the bulk band gap were performed using a homebuilt setup. The relative composition of the CZTS films were analyzed by XRF spectroscopy using a PANalytical Epsilon 5 EDXRF spectrometer.

TEM measurements were performed using a probe corrected FEI Titan Themis equipped with the SuperX system for energy-dispersive X-ray spectroscopy (EDS) and operated at 200 kV. A focused ion beam and a scanning electron microscope (FEI Strata DB235) were employed to prepare the cross-section TEM lamellae.

#### X-ray Spectroscopy

XPS measurements including core level spectroscopy and valence band (VB) spectroscopy were conducted using synchrotron radiation under variable photon energies (from soft to hard X-rays) to monitor the composition, chemical state, and electronic structure of CZTS-based solar cells.

HAXPES measurements were performed at both the GALAXIES beamline at the Soleil synchrotron (France)^16^ and at the I09 beamline at the Diamond Light Source (UK)^17^ to gain information about the chemical and electronic properties of CZTS and ZTO/CZTS. The photoemission spectra for the ZTO/CZTS interface measurements were performed at GALAXIES employing a VG Scienta EW4000 energy electron analyzer at normal emission and excitation energies of *h*ν = 3 keV and *h*ν = 9 keV, respectively. A pass energy of 200 eV yielding an analyzer resolution of 150 meV was used for all measurements, and the binding energy was calibrated by measuring the 4f spectrum of a grounded clean Au foil and setting the Au 4f_7/2_ binding energy to 84.0 eV, if not otherwise mentioned in the text. XPS line intensities were quantified by fitting them with Voigt profiles and a linear background using Igor Pro software and taking into account the respective values for the inelastic mean free path^18,19^ and photoionization cross-section^20,21^ including the asymmetry parameters of photoelectric angular distributions.^21,22^ The analyzer transmission function has not been taken into account, which may introduce an error in the calculation of the absolute values, but the aim of this study is to compare relative amounts between the investigated samples, and the method was therefore found to give reliable results, as we previously discussed.^8,23^

The depth profile of CZTS anneal measurements were performed using HAXPES at beamline I09 (Diamond) by exposing the sample to 1 × 10^–5^ mbar N_2_ at temperatures between 100 and 200 °C for 30 min (including warming up and cooling time). Photoemission spectra were recorded before and after the N_2_ anneal treatment employing a VG Scienta EW4000 energy electron analyzer and both soft and hard X-rays. The photon energy was varied between 1100 and 7050 eV, which allowed for varying the information depth. A defocused beam was used to minimize the radiation damage, and no evidence of beam damage was observed.

XAS measurements were performed at the P64 beamline at the Petra III synchrotron radiation facility in Germany.^24^ The cation K-edges (Cu, Zn, and Sn at 8.98, 9.66, and 29.2 keV, respectively) were measured in the fluorescence mode at both 10° (bulk sensitive) and 0.5° (probing depth ∼ 150 nm) incidence angles using a Ge detector. All measurements were performed in air and at room temperature. Metal foils measured simultaneously with the samples were used for energy calibration. The XAS measurements included both the X-ray absorption near-edge structure and extended X-ray absorption fine structure (EXAFS) regions. Athena software, part of the Demeter package,^25^ was used for preprocessing and analysis of the EXAFS data. Preprocessing of data included alignment, edge calibration, deglitching, normalization, and background subtraction. The energies at the Cu, Zn, and Sn K edges were determined by the first inflection point of the corresponding absorption edge data characterizing the reference Cu, Zn, and Sn foils, respectively, calibrated to the reported energies.

## Results and Discussion

### Electrical Properties of Cu_2_ZnSnS_4_-Based Thin Film Solar Cells with an ALD Buffer

**Figure 1:**
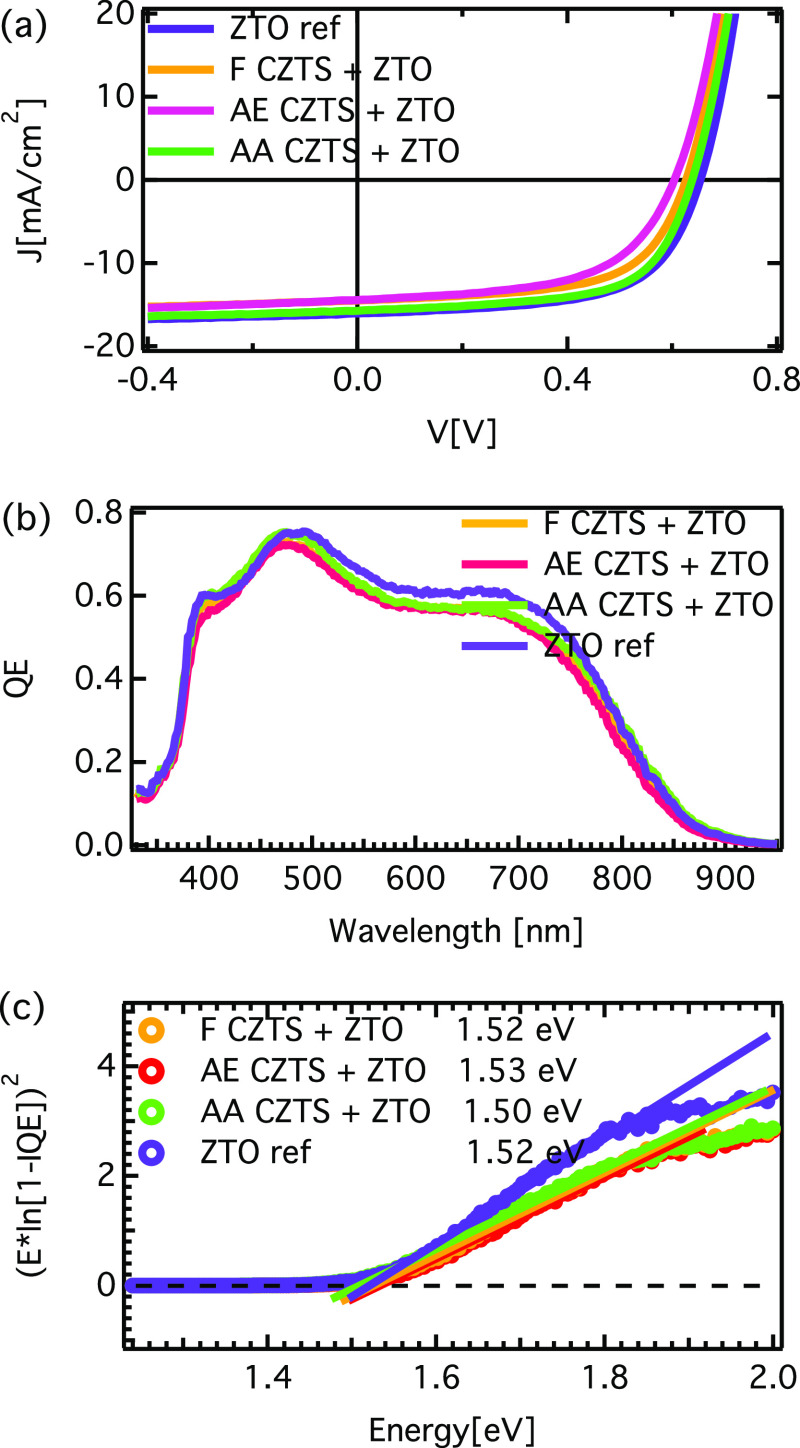
(a) *I*–*V* characteristics of the best cell from reference ZTO/CZTS device (1000 cycles at 120 °C) and surface-treated CZTS + ZTO devices (700 cycles at 120 °C). (b) Quantum efficiency measurements of the investigated ZTO/CZTS samples. (c) Determination of the band gaps from an extrapolation of the leading edge to the extended baseline in the spectrum, as discussed more in ref (11). The following notation is used: F = fresh, AE = air exposed, and AA = air annealed.

The *I*–*V* characteristics for the surface-treated CZTS + ZTO devices prepared from the samples investigated in this work are presented in Figure S1. The set *I–V* results show clear differences, with air annealing giving the best results, and only the best cell values are used in the discussion. The device results of the best cell from reference CZTS devices and surface-treated CZTS with an ALD ZTO buffer are shown in Figure 1a, and the main parameters for the best solar cell for each sample are summarized in Table 1. In a comparison between cells having different ZTO thicknesses, the ZTO reference sample (1000 cycles) gives the highest efficiency among the investigated samples, indicating that thicker ZTO gives better performing devices. However, optimization of the ZTO buffer layer is not the aim of the present work, and thus, thinner buffer layer samples (700 cycles, ∼20 nm on CZTS according to TEM) were studied to be able to investigate the buffer/absorber interface directly and non-destructively by employing depth-resolved photoemission spectroscopy (i.e., HAXPES), as described in more detail below. A comparison with the recently reported data on the CdS/CZTS^8^ reference from similar CZTS depositions shows an increased device performance when ALD ZTO is used as a buffer layer (non-treated CZTS absorber). The *V*_oc_ increase for ZTO/CZTS is likely related to improved band alignment between the buffer and absorber, as previously discussed for ALD buffers.^10^

**Table 1:** Photovoltaic Properties of the Solar Cells Fabricated from the Set of Samples Discussed in This Work (Cell Structure: Al:ZnO/CdS/CZTS/Mo/SLG)^a^

sample ID	comment	η	*J*_sc_	*V*_oc_	FF
CdS ref	reference thick CdS on fresh CZTS^8^	5.3	14.8	610	58.6
ZTO ref	reference thick ZTO (1000 cycles at 120 °C) on fresh CZTS	6.5	16.1	653	61.9
F	fresh CZTS + ZTO (700 cycles at 120 °C)	5.6	14.5	626	61.3
AE	24 h AE CZTS + ZTO (700 cycles at 120 °C)	5	14.4	604	57
AA	AA CZTS + ZTO (700 cycles at 120 °C)	6.3	15.7	638	63.2

a The best cell data are reported for each sample. For comparison, data from the CdS/CZTS reference, as previously reported,^8^ are included. (η: conversion efficiency [%], *J*_sc_: short-circuit current density [mA/cm^2^], *V*_oc_: open-circuit voltage [mV], and FF: fill factor [%]).

Comparing the influence of the CZTS surface treatment on the device properties, it is clear that an air annealing treatment (AA) resulted in increased device properties as compared to that of a non-treated (F) CZTS when similar ZTO films are deposited (700 cycles at 120 °C yielding a similar XRF composition), in agreement with previous reports for CdS/CZTS.^8^ However, the AE CZTS sample shows the lowest device performance when the ZTO buffer is employed, in contrast to our previous work using CdS buffer where the efficiency of the surface-treated CZTS devices has been found to increase with the surface treatment of CZTS (F < AE < AA).^8^ No significant differences between the differently treated absorbers are observed for the bulk band gap values, as determined from the quantum efficiency measurements (Figure 1b,c).

### Properties of Surface-Treated Cu_2_ZnSnS_4_ and the Influence on the ALD Buffer Layer Growth

#### TEM Measurements of Cu_2_ZnSnS_4_-Based Thin Film Solar Cells with ALD Buffer

**Figure 2:**
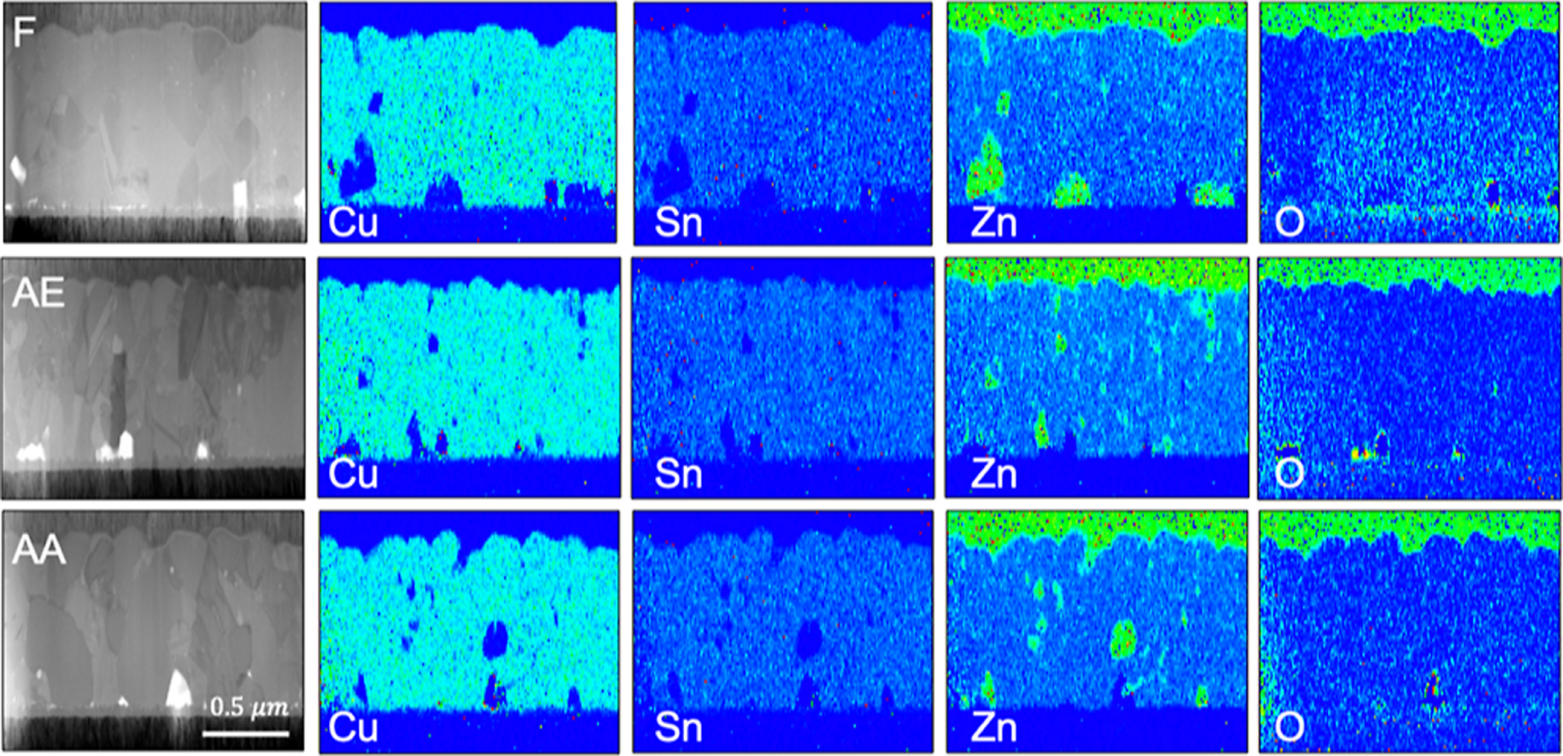
Cross-sectional TEM and EDS measurements of the fresh (F, top row), AE (middle row), and AA (bottom row) samples investigated. In the EDS measurements, red represents a high relative concentration, while blue represents a low relative concentration.

Cross-sectional TEM measurements were performed to obtain information about the morphology and composition profile of the investigated samples. Figure 2 shows the cross-sectional TEM analysis along with EDS measurements of the F, AE, and AA CZTS samples after device formation with ALD ZTO and i:ZnO/Al:ZnO window layers. The TEM analysis showed no clear difference with respect to the ZTO buffer layer between the surface-treated and non-treated CZTS samples, and a similar buffer layer thickness was observed for all samples (∼20 nm on CZTS, see Figure S2, Supporting Information). However, small local variations are observed in the bulk of the differently treated CZTS absorbers. Slightly smaller grains are observed for the AE and AA samples than that of the F sample. Further, Zn-rich regions are observed in all samples (likely ZnS phases), which are larger and located toward the back contact for the F sample. Previously, changes in the distribution of ZnS were reported for low-temperature annealing of CZTS,^26^ and it cannot be excluded that the formation of ZnS phases may also impact the device properties in the present work.

#### Ex Situ N_2_ Annealing of CZTS Studied by XPS/HAXPES

In a related study, we investigated the effect of the air annealing treatment on the CZTS surface, and the results showed the formation of SnO_*x*_ species on the CZTS surface, which were found to not be removed by the KCN etching step applied prior to CdS buffer layer deposition.^8^ As identical CZTS surface treatments were employed in this work followed by KCN etching, similar results are expected (i.e., SnO_*x*_ formation upon CZTS surface treatment). However, in contrast to the CdS growth by chemical bath deposition, the ALD process consists of an additional step where samples are annealed in N_2_ at the deposition temperature (120 °C for 30 min in this work). We have thus investigated how such an annealing treatment may influence the surface properties of a non-treated (fresh) CZTS absorber, which may impact the buffer layer growth. For this purpose, a N_2_ annealing study has been conducted, which mimics the treatment before the ALD buffer layer growth. The annealing and subsequent measurements were performed under vacuum conditions (without exposing samples to the air after the treatment) and temperatures between 100 and 200 °C (for 30 min including heating up and cooling times) by employing XPS with different probe depths. Both the more surface-sensitive (XPS, photon energy of 1.1 keV) and more bulk-sensitive, below the CZTS surface (down to ∼30 nm by HAXPES, photon energies of 2.35 and 7.05 keV) measurements, were performed as discussed further below.

**Figure 3:**
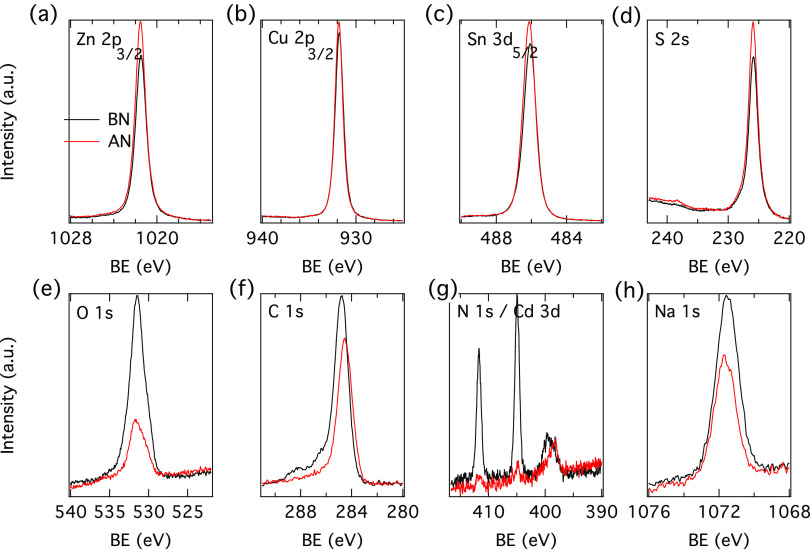
Photoemission spectra before and after the N_2_ annealing treatment of CZTS recorded with an excitation energy of 2.35 keV: (a) Zn 2p_3/2_, (b) Cu 2p_3/2_, (c) Sn 3d_5/2_, (d) S 2s, (e) O 1s, (f) C 1s, (g) N 1s, and (h) Na 1s. The spectra have been aligned to the Zn 3d peak (set to 10.0 eV). The following notation is used: BN = before N_2_ annealing and AN = after N_2_ annealing.

The survey spectra for all photon energies employed are shown in Figure S3 (Supporting Information), and the core level spectra are shown in Figure 3 (2.35 keV) and Figure S4 (1.1 keV) and Figure S5 (7.05 keV) in the Supporting Information. Since no other calibration was available, the spectra were calibrated to Zn 3d to facilitate a relative comparison between the samples. As the 7.05 keV survey spectra did not show any difference before and after the N_2_ annealing, the core level spectra after the annealing were omitted at this energy.

As expected, the spectra recorded before the annealing show peaks from the CZTS absorber as well as some C and O contamination (weaker at higher photon energies). In addition, some F (fluorine) and Cd contamination is observed before the annealing for the spectra recorded with 1.1 and 2.35 keV photon energies and is likely from the KCN solution, which is commonly used in our lab to etch different CZTS absorbers. After the N_2_ annealing, no F signal is observed, the C and O contamination decreases, and only traces of Cd are observed, suggesting that the N_2_ annealing below 200 °C helps to “clean” the CZTS surface. The mechanism for the decrease in Cd is unclear, but most probably, it diffuses into the bulk of the material. Even though some previous studies reported on the effect of Cd addition to the solar cell behavior, the aim of this study is to compare the effect of different surface treatments on samples that shall have the same level of contaminants (including Cd). The very small amount of Cd after the N_2_ annealing step in this work would indicate its reduced influence for the ZTO/CZTS solar cells, in contrast to the chemical bath deposition of CdS buffer, which does not contain a N_2_ annealing step. Besides some intensity changes, no clear differences are observed in the cation core level spectra (no binding energy shifts, assuming a similar Zn chemical environment) before and after the N_2_ annealing at both employed energies, suggesting that the chemical environment around the absorber elements is not significantly altered.

The 1.1 and 2.35 keV measurements show similar trends, and thus, only the 2.35 keV measurements are further discussed below. The core level spectra reveal that the main CZTS signals (Zn, Sn, and S) increased after the N_2_ annealing and the Cd signal decreased, likely due to the cleaning of the surface. However, the Cu signal remains almost constant, while for the measurements performed at 1.1 keV, it decreases after the N_2_ annealing, and it is thus not excluded that the surface composition may change during the N_2_ annealing, as described in more detail below. Compositional changes are likely to influence the device performance. The C 1s core level spectra show mainly the contribution from adventitious carbon (∼285 eV), and some weak signals from CO_*x*_ species are seen on the spectra recorded before the annealing as a broad shoulder (below 290 eV) on the high binding energy side of the main peak. Carbonates may form on the CZTS surface, as previously reported for AE CZTS.^7^

After the N_2_ annealing, we do see a concomitant decrease of the CO_*x*_ species as well as O 1s and Na 1s signals, which may indicate that Na_2_CO_*x*_ and Na_2_O species were likely present at the near CZTS surface before the annealing, which then were reduced during the N_2_ annealing. Further, the concomitant increase of the Zn, Sn, and S and decrease of Na 1s, O 1s, and CO_*x*_ peaks would suggest that Na_2_CO_*x*_ and Na_2_O compounds were likely present at the CZTS surface before the N_2_ annealing. In addition, the S 2s core level spectrum shows the appearance of some weak sulfite/sulfate species (@237–238 eV) after the N_2_ annealing in addition to the sulfide species (S 2s main peak @226 eV). Since the Na_2_SO_*x*_ species are observed after the N_2_ annealing, it is likely that they were present on the CZTS surface before the annealing (underneath the CO_*x*_ species) and are not removed by the applied N_2_ annealing or that they were formed during the N_2_ annealing. Na-containing surface compounds, Na–SO_4_ and Na–CO_3_, were previously observed on the surface of CZTS that was exposed to air and were found to be removed by a KCN etching treatment.^7^ According to the above-mentioned study, Na_2_S, Na_2_SO_4_, Na_2_CO_3_, Na_2_SO_4_, and Na_2_CO_3_ hydrate compounds have the lowest negative Gibbs energy of formation among all possible compounds on the CZTS surface. It is thus likely that some of these species are also present on the CZTS sample in this study. Even though we do not have data on how the removal of Na–CO_*x*_ or conversion into Na–SO_*x*_ compounds takes place (as N_2_ is an inert gas) and considering that it is unlikely that the sodium salts desorb below 200 °C, it is suggested that the sodium ion either diffuses in the grain boundaries within the sample or leaves as a separate element. Previously, Na segregation (from the glass/Mo back contact) has been observed based on ex situ studies, with Na and S evaporation at high temperature or low pressure.^27^ Thus, it is likely that Na evaporation (high *T* and low *P*) occurs during the N_2_ annealing treatment employed in this work. As the Na-S(-O) species are similar in Na 1s and also as the Na–sulfide peak in S 1s is not easy to discern from other Me–S species, it is difficult to distinguish any Na_2_S species from the XPS spectra. Thus, we cannot exclude that Na_2_S species are also present on the CZTS surface.

Increasing the photon energy to 7 keV allowed us to probe deeper into the CZTS absorber (probing depth ∼ 30 nm). The measurements before the annealing (Figure S5, Supporting Information) reveal the main signals from Cu, Zn, Sn, and S, as expected, and some weak Na, O, and C signals. No signals from CO_*x*_ or SO_*x*_ are observed at this energy, and it is expected that the observed changes on CZTS are limited to the near-surface region (down to about 10 nm).

In addition, the results show that the CZTS near-surface composition changes upon the N_2_ annealing treatment. The relative composition analysis performed at all employed photon energies is given in Figure S6. Cu depletion and Zn and Sn enrichment are observed after the N_2_ annealing treatment at the CZTS near-surface region (for both 1.1 and 2.35 keV measurements). This, together with the removal of NaCO_*x*_ species from the CZTS surface, may partly explain the observed positive impact on the solar cell behavior for ALD buffer as compared to CdS. The shallow core level spectra shown in Figure S7 indicate that the Na species are enriched at the surface, as indicated for both the 1.1 and 2.35 keV measurements before the N_2_ annealing.

Further, the VB spectra were recorded at 1.1 and 2.35 keV in order to obtain information on how the electronic properties may change upon the N_2_ annealing treatment. From the results shown in Figure S8, we cannot observe any clear differences in the valence band maximum position for the cases before and after the N_2_ annealing treatment at both employed energies, indicating that the general electronic properties (VB alignment) are very similar.

#### Investigation of Bulk Properties by XAS

To investigate if bulk properties may be responsible for the observed changes in device performance between the AA and non-treated CZTS samples, XAS data were collected at the cations K edges. Depth profiling possibility, by changing the incidence angle of the X-rays by XAS (10 and 0.5° employed in this work), offers opportunities to observe compositional changes below the CZTS surface and further into the bulk (probing depth ∼ 150 nm for 0.5° measurements vs ∼2000 nm for the 10° measurements). The raw XAS spectra recorded at 10 and 0.5° for the Zn, Cu, and Sn K edges are shown in Figure S9a–c (Supporting Information). EXAFS data collection was performed to gain information about the local structure surrounding the Zn, Cu, and Sn elements. The Fourier transforms of the Zn, Cu, and Sn K edges EXAFS spectra of the non-treated and AA CZTS + ZTO samples are presented in Figure S9d–h (Supporting Information), where *R* represents the radial distance from the absorbing atom. A comparison of the intensity and the shape of the Fourier transform data shown in Figure S9d–h shows that the bulk coordination structure on a sub-nanometer scale (incidence angle of 10°) is largely unaffected by the surface treatment of CZTS (F or AA) or the deposition of ZTO buffer, and only Me–S bonds are observed in the Fourier transform data. Further, EXAFS spectra at the Cu and Zn K-edge measurements at a 0.5° incidence angle (corresponding to a probing depth of about ∼150 nm) indicate an enrichment of Zn–S toward the surface for the AA sample as compared to that for the non-treated sample, whereas the Cu–S content remains more or less constant (Figure S9g–h), which may, in part, explain the increased device performance for the AA sample as compared to that for the non-treated (F) sample.

#### Chemical and Electronic Properties of the ZTO/CZTS Interface for Surface-Treated CZTS Studied by HAXPES

To obtain information on the chemical and electronic properties of the ZTO/CZTS interface, HAXPES measurements were employed. Figure S10 (Supporting Information) shows the survey spectra of the non-treated and surface-treated CZTS + ZTO samples using a photon energy of 3 keV. For comparison, spectra recorded from reference samples (CZTS and ALD) are also included. The survey spectra are very similar for all CZTS + ZTO samples and show mainly signals from the Zn–Sn–O buffer layer. In addition, some weak C contamination is observed, and no absorber signals (Cu or S) were detected since the probing depth at this photon energy (3× IMFP ∼ 12 nm) is smaller than the buffer layer thickness on CZTS (∼20 nm). No significant differences are observed between the non-treated and surface-treated CZTS + ZTO samples, as also supported by the high-resolution core level spectra (see Figure S11, Supporting Information), suggesting that the near-surface ZTO layer is not affected by the CZTS surface treatment. The O 1s spectra for all samples show a component at 530.5 eV (green), representative of a metal oxide (Me–O_*x*_) component, that is, SnO_*x*_ and ZnO_*x*_, in addition to the hydroxide component (blue component at around 532 eV), as was previously observed on CZTS.^8^ Since water pulses are used during the ZTO ALD process, it is very likely that the deposited ZTO contains hydroxide species at these low deposition temperatures.

**Figure 4:**
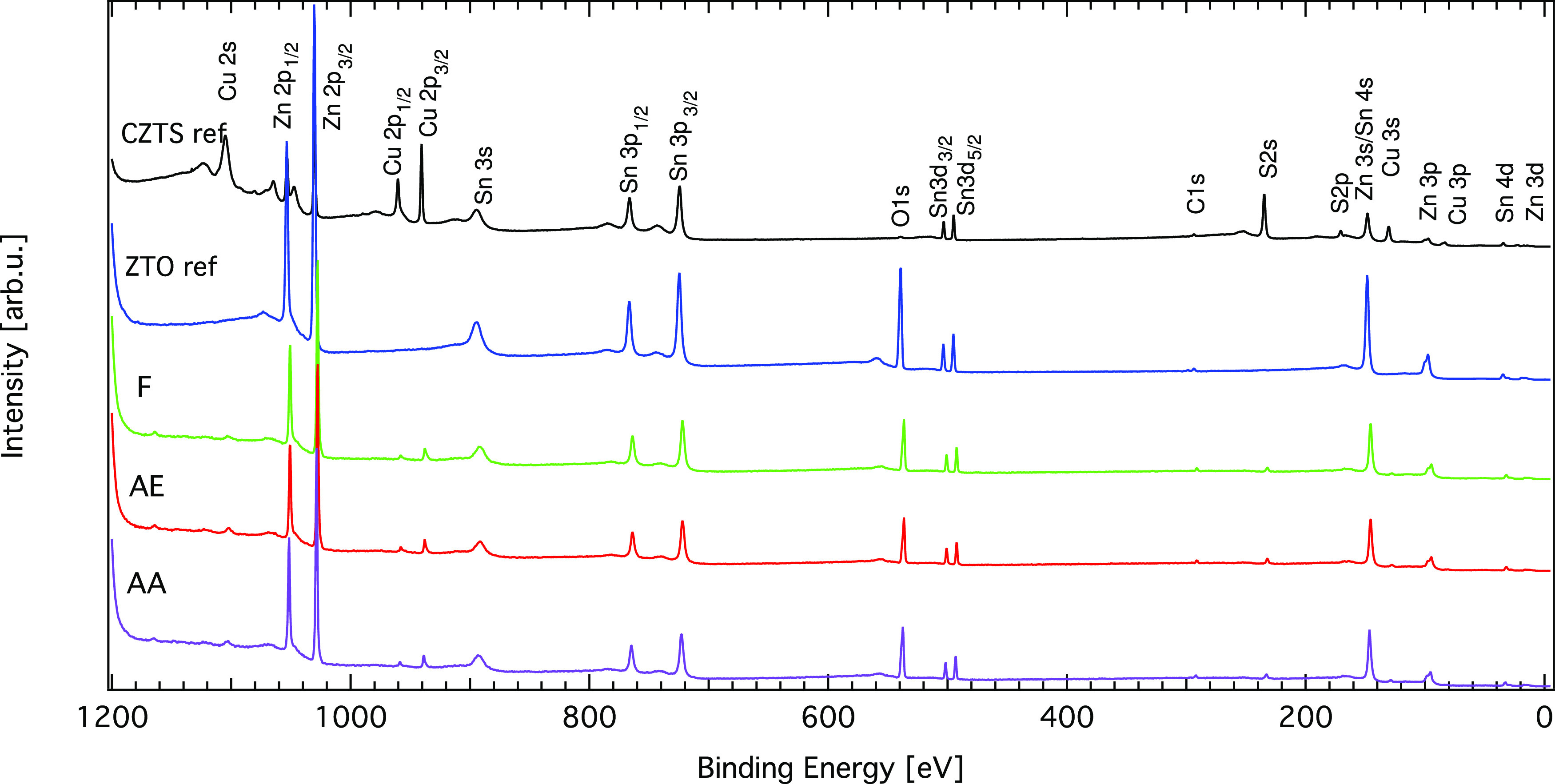
HAXPES survey spectra recorded at 9 keV for the ALD ZTO on non-treated (F) and surface-treated CZTS samples (AE and AA) as well as reference samples (ZTO and CZTS ref), recorded with an excitation energy of 9 keV. The most prominent lines are labeled, and the spectra have been vertically offset for clarity. The following notation is used: F = fresh, AE = air exposed, and AA = air annealed.

Upon increasing the photon energy to 9 keV, clear signals from the absorber (Cu and S) are visible on the spectra of the CZTS + ZTO samples together with Zn, Sn, and O signals (Figure 4), indicating that both ZTO and CZTS near-surface regions are probed at 9 keV. The corresponding high-resolution spectra (Figure S12, Supporting Information) of individual core levels show no significant differences between the non-treated and surface-treated CZTS + ZTO samples, except for some intensity changes, as discussed below. Two components were used to fit the Sn 3d and Zn 2p core levels (representing Me–S and Me–O chemical species) for the CZTS + ZTO samples as a broadening of the core levels was observed as compared to that for the CZTS reference (see Table S1, Supporting Information) and as also suggested by the presence of the Cu and S signals that both Me–S and Me–O chemical species shall be present at this photon energy (IMFP for these core levels in ZTO is similar at 9 keV and ranges from 12 to 13 nm). The O 1s spectra are very similar for the CZTS + ZTO samples and are also similar to the spectra recorded at 3 keV, and there is no clear evidence of an O-containing layer below the buffer for the treated samples, at least within the probing depth (∼30–35 nm at 9 keV). However, since it is not easy to distinguish between O species from the ZTO layer or O species that may exist at or below the ZTO/CZTS interface (all Me–O species), we cannot rule out the existence of an O-containing interlayer at the ZTO/CZTS interface for the surface-treated CZTS samples, as previously observed for CdS/CZTS (i.e., SnO_*x*_)^8^ or as previously reported to be beneficial for the device performance [i.e., Zn(O,S) layer formation.^9^ Also, any Me–O species that may exist at the CZTS surface from the surface treatment might be masked by the higher signal from the ZTO layer.

In addition, the shape and broadness of the absorber (Cu and S) and buffer (Zn, Sn, and O) signals do not change between the non-treated and surface-treated CZTS + ZTO samples as Voigt profiles with identical relative Gaussian and Lorentzian widths for a particular line were used to fit the spectra at each photon energy (3 and 9 keV) (see Figures S11 and S12 and Table S1, Supporting Information). This suggests that the chemical state around the absorber or buffer elements is not significantly altered by the CZTS surface treatment. Further, no significant binding energy shifts have been observed for the surface-treated versus non-treated CZTS + ZTO samples, supporting the assumption of a similar chemical environment around the absorber elements between the investigated samples.

**Figure 5:**
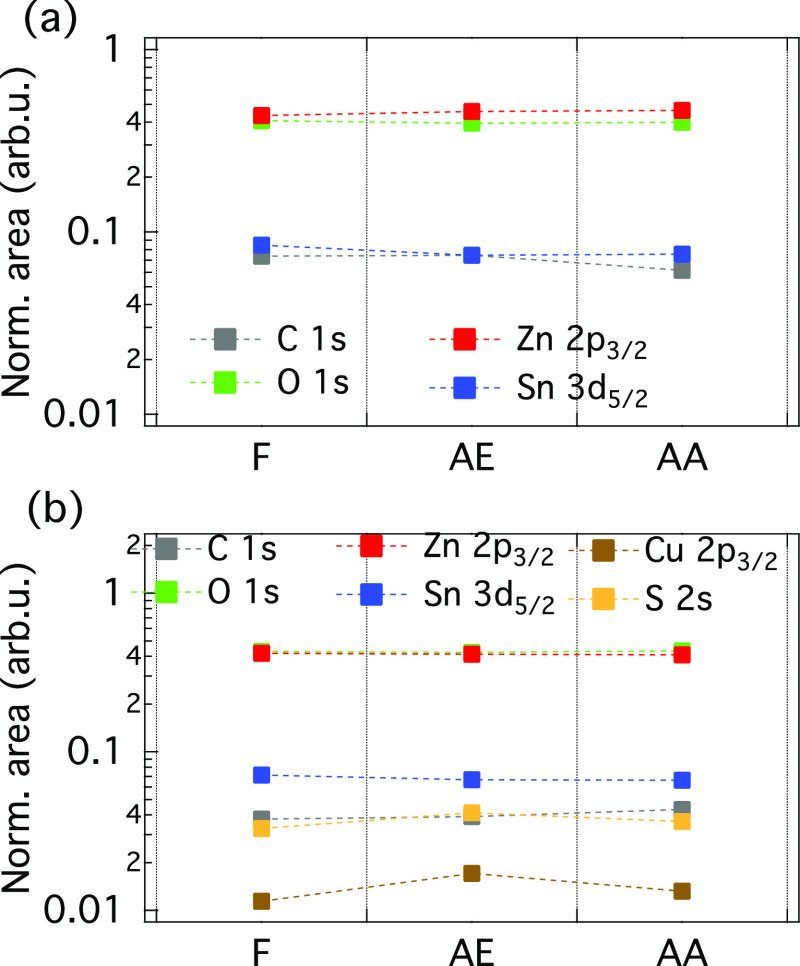
Evolution of the C 1s, O 1s, Zn 2p_3/2_, Sn 3d_5/2_, S 2s, and Cu 2p_3/2_ core level spectra for the investigated ZTO/CZTS samples exposed to different surface treatments at an excitation energy of 3 (a) and 9 keV (b), respectively. The peak areas were normalized such that the area sum of all shown photoemission lines is 1 for each sample. Note that a logarithmic *y*-scale has been employed to better visualize small contributions, and dotted lines are only used to guide the eyes. The following notation is used: F = fresh, AE = air exposed, and AA = air annealed.

To compare the influence of the CZTS surface treatment and buffer layer growth at the buffer/absorber interface region, XPS intensity changes have been compared for the investigated samples. Since both the absorber and buffer contain Zn and Sn elements, it is difficult to distinguish between buffer- and absorber-related peaks for Zn and Sn as these elements exist in both layers. Even more, a surface treatment may change the CZTS near-surface composition, and it is thus not straightforward to draw any conclusion regarding the formation of an interlayer, as previously observed.^8^Figure 5 shows the attenuation behavior of the different core levels recorded using excitation energies of 3 (a) and 9 keV (b), respectively. The signal using the peak area for the different components is corrected for IMFP and cross-section (including asymmetry). In addition, the peak areas were normalized such that the sum of all shown photoemission lines is 1 for each sample. Please note that the overall peak areas have been considered in Figure 5b, whereas Figure S13 contains the deconvoluted components of Sn and Zn core levels (i.e., Me–S and Me–O chemical states). No significant compositional changes are observed between the investigated samples, except a slightly increased Cu content for the AE sample. Note that the compositional changes on a thin interfacial layer may not be visible with HAXPES due to limited sensitivity.

#### Discussion: Surface Treatments of the Cu_2_ZnSnS_4_ Absorber and the Influence on the ALD Buffer Layer Growth

In our recent work,^8^ we showed that a CZTS surface treatment (air annealing or air exposure) was found to influence the buffer layer growth and a thicker CdS overlayer was observed for the surface-treated CZTS as compared to that for the non-treated CZTS. In this case, the efficiency of the surface-treated CZTS devices has been found to increase with the surface treatment of CZTS (F < AE < AA) and also correlated to the CdS overlayer growth and the presence of interface species (i.e., SnO_*x*_) for surface-treated CZTS (AE and AA) samples.

Similarly, when a ZTO buffer layer has been employed, an increased performance is observed for the AA CZTS sample as compared to that for a non-treated (F) sample, which may suggest that chemical changes at the CZTS surface upon air annealing (i.e., SnO_*x*_ formation) may influence the ZTO/CZTS interface and the ZTO buffer layer growth. In order to rule out other variables that may influence the device performance such as variation in the CZTS bulk composition or buffer layer thickness, bulk-sensitive XAS and cross-sectional TEM were performed. XAS results show no significant differences between the AA and non-treated (F) samples, thus suggesting that the bulk properties are very similar. Further, no clear differences in the ZTO buffer layer thickness are observed depending on the CZTS surface treatment, as determined by TEM. However, some compositional changes are observed toward the CZTS surface region for the AA CZTS + ZTO sample (increased Zn and Sn content and decreased Cu content), which may be linked to the increased device performance.

As the ALD deposition of the ZTO buffer contains an additional N_2_ annealing step, which may further impact the CZTS surface and buffer layer growth, a N_2_ annealing study that mimics the low-temperature annealing of CZTS prior to ALD of oxide films has been performed in order to understand how the absorber surface properties may change during such a treatment. The results indicate that the N_2_ annealing step before the ALD buffer layer deposition, which removes Na–CO_*x*_ or Na_2_O species from the CZTS surface, may be useful for device performance.

Previously, Xie et al.^27^ highlighted the importance of appropriate Na distribution at the CZTSSe/CdS interface for device performance: Na accumulation at the interface was linked to severe degradation of device performance, while a Na-deficient interface has a positive impact on the device performance. In the current work, we are not able to detect any Na or Na-containing species that may be present at the ZTO/CZTS interface and that can affect device performance. However, we cannot exclude that interlayer species in small amounts are present at the ZTO/CZTS interface, which cannot be detected using HAXPES due to its limited sensitivity. The samples are always exposed to air for a short period of time (<5 min) before being loaded into the ALD reactor. During this short air exposure, carbonate can continuously form and sulfide phase may be transformed to sulfite and sulfate phases on the CZTS surface.^7^

Further, by employing HAXPES, we studied how a surface treatment of the CZTS absorber (air exposure or air annealing) may influence the chemical and electronic properties at the ZTO/CZTS interface and device properties. For comparison, measurements for non-treated CZTS are also included. Within the limitations of this method to detect small concentrations/thin interlayer formation at higher photon energies, the results show that the chemical properties at the ZTO/CZTS interface are not significantly affected by the CZTS surface treatment.

The photoemission and absorption spectroscopy results provide evidence that the near CZTS surface composition changes with its surface treatment and that the N_2_ annealing treatment prior to ALD buffer layer growth may change the absorber surface chemistry and composition. It is thus possible that the initial ALD composition and properties are influenced by any change in the substrate surface reactivity toward the ALD precursors. A previous work by our group shows that sodium salts may inhibit the ALD growth. In ref (28), it is shown that this can result in quite significant ZTO composition differences during the first nanometers of growth, likely due to the different nucleation times between the different sub-cycles. Thus, the presence of SnO_*x*_ species at the CZTS surface for the AA sample in this work together with the compositional changes (at the CZTS near-surface region) and different CZTS surface chemistry, which may influence the initial ZTO composition, could lead to an improved device performance as compared to that of the non-treated sample.

## Conclusions

Increased device performance is observed when an ALD ZTO buffer layer is used for CZTS thin film solar cells as compared to CdS, and the performance is further increased after an air annealing treatment of the absorber. However, no significant differences are observed in the bulk or in the ZTO buffer layer thickness for different surface treatments of the CZTS absorber, and some compositional changes are observed toward the CZTS surface. Formation of SnO_*x*_ species before the buffer layer growth, together with Zn enrichment for the AA CZTS + ZTO sample, may explain the improved device performance. Even more, the results indicate that the N_2_ annealing treatment typically applied prior to the ALD buffer layer deposition is likely to change the CZTS near-surface chemistry and composition differently for differently treated CZTS absorbers. Thus, a re-optimization of the ALD buffer layer growth for different CZTS surface treatments may be required.

## Supporting Information Available

Additional graphics of device performance, cross-sectional TEM, N_2_ annealing study of CZTS, XAS analysis, HAXPES analysis, and the changes in the full width at half maximum of the main absorber core levels (PDF)
