# Supplementary material for: Atomic Layer Grown Zinc–Tin Oxide as an Alternative Buffer Layer for Cu2ZnSnS4-Based Thin Film Solar Cells: Influence of Absorber Surface Treatment on Buffer Layer Growth
Source: ACS Appl Energy Mater. 2022 Oct 14;5(11):13971–80. doi: 10.1021/acsaem.2c02579 (PMC9710524; doi:10.1021/acsaem.2c02579)
Supplement: Supplementary file 1 — ae2c02579_si_001.pdf [file ae2c02579_si_001.pdf]

Supporting Information

Atomic layer grown zinc-tin-oxide as alternative buffer layer for Cu_2ZnSnS_4-based thin film solar cells: influence of absorber surface treatment on buffer layer growth

Natalia M. Martin,[*,†] Tobias Törndahl,[†] Melike Babucci,[†] Fredrik Larsson,[†,‡] Konstantin Simonov,[¶,§] Dorotea Gajdek,[‖] Lindsay R. Merte,[‖] Håkan Rensmo,[¶] and Charlotte Platzer-Björkman[†]

†*Solar Cell Technology, Department of Materials Science and Engineering, Uppsala University, SE-751 21 Uppsala, Sweden*

‡*EVOLAR AB, Uppsala, 756 51, Sweden*

¶*Molecular and Condensed Matter, Department of Physics and Astronomy, Uppsala University, SE-751 21 Uppsala, Sweden*

§*Swerim AB, Department of Materials and Process Development, Box 7047, SE-164 07 Kista, Sweden*

‖*Department of Materials Science and Applied Mathematics, Malmö University, SE-211 19 Malmö, Sweden*

E-mail: Natalia.Martin@angstrom.uu.se

Figure S1 shows the I-V characteristics for the surface treated CZTS + ZTO devices prepared from the samples investigated in this work.

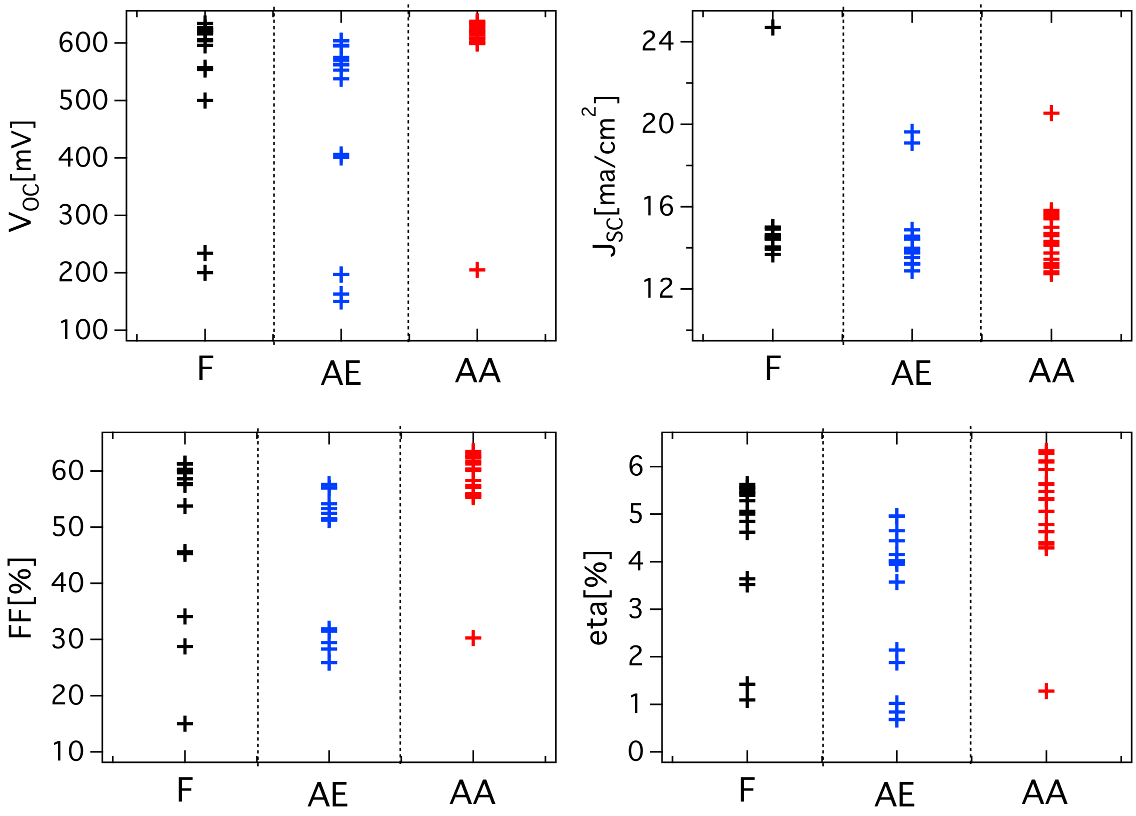

Figure S1: Photovoltaic properties of the ZTO/CZTS samples investigated in this work: V_{OC}, open-circuit voltage [mV]; FF, fill factor [%]; J_{SC}, short-circuit current density [mA/cm^2]; eta, conversion efficiency [%]) The following notation is used: F=Fresh, AE=Air Exposed, AA=Air annealed.

Figure S2 shows a zoom in the cross-sectional TEM images recorded from the investigated samples indicating a constant buffer layer thickness.

Figure S3 and Figures S4, S5 show the survey and high resolution photoemission spectra for the CZTS sample before and after the N_2 anneal treatment recorded using 1.1, 2.35 and 7.05 keV photon energy as indicated.

Figure S6 shows the relative composition analysis for the CZTS sample before and after the N_2 anneal treatment as determined from the photoemission measurements.

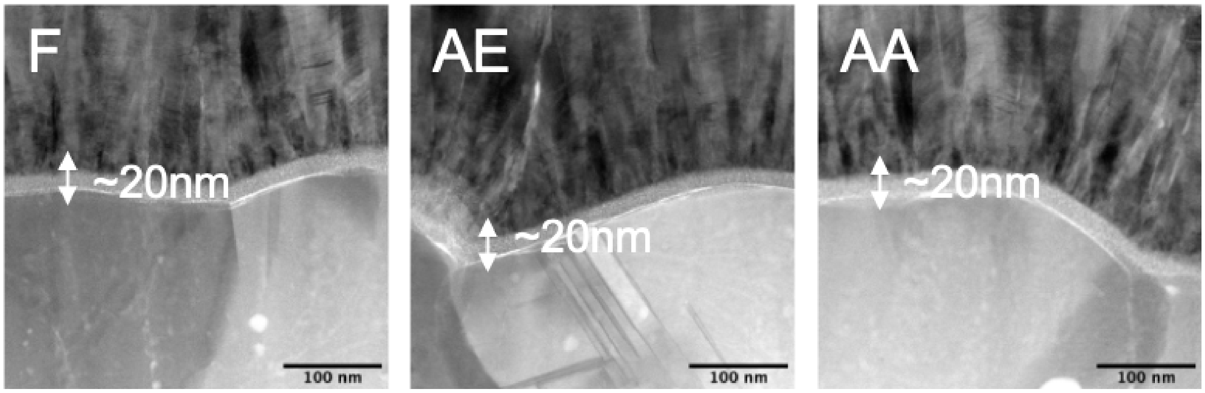

Figure S2: Zoom in TEM images of the fresh (F, left), air exposed (AE, middle) and air annealed (AA, right) CZTS + ZTO samples investigated. The arrows indicate the ZTO buffer layer thickness which was found to be similar for all air treatments of CZTS.

Figure S7 shows the extended VB spectra including shallow core levels for the CZTS sample before and after the N_2 anneal treatment recorded with excitation energies of 1.1 keV, 2.35 keV and 7.05 keV, respectively.

Figure S8 shows the VB spectra for the CZTS sample before and after the N_2 anneal treatment recorded with excitation energies of 1.1 keV and 2.35 keV, respectively.

Figure S9 shows the raw XAS spectra (a-c) and Fourier transforms of Zn, Cu and Sn K edges EXAFS spectra of the non-treated and air annealed CZTS + ZTO sample recorded at incidence angles of 10 degrees (d-f) and 0.5 degrees (g-h) as indicated.

Figure S10 and Figure S11 show the survey and high resolution photoemission spectra with the corresponding peak fits of the investigated sample series recorded using 3 keV photon energy. At this energy only the ZTO buffer layer is probed for all samples.

Figure S12 shows the high resolution photoemission spectra with the corresponding peak fits of the investigated sample series recorded using 9 keV photon energy. At this energy both the CZTS and ZTO are probed as indicated by the peak assignment. To fit the spectra, a linear background and Voigt profiles of identical Gaussian and Lorentzian widths for a particular line were employed. The artefact in Cu 2p (broad component at lower binding energy), also observed for CZTS ref sample is likely an Auger peak and needs to be further

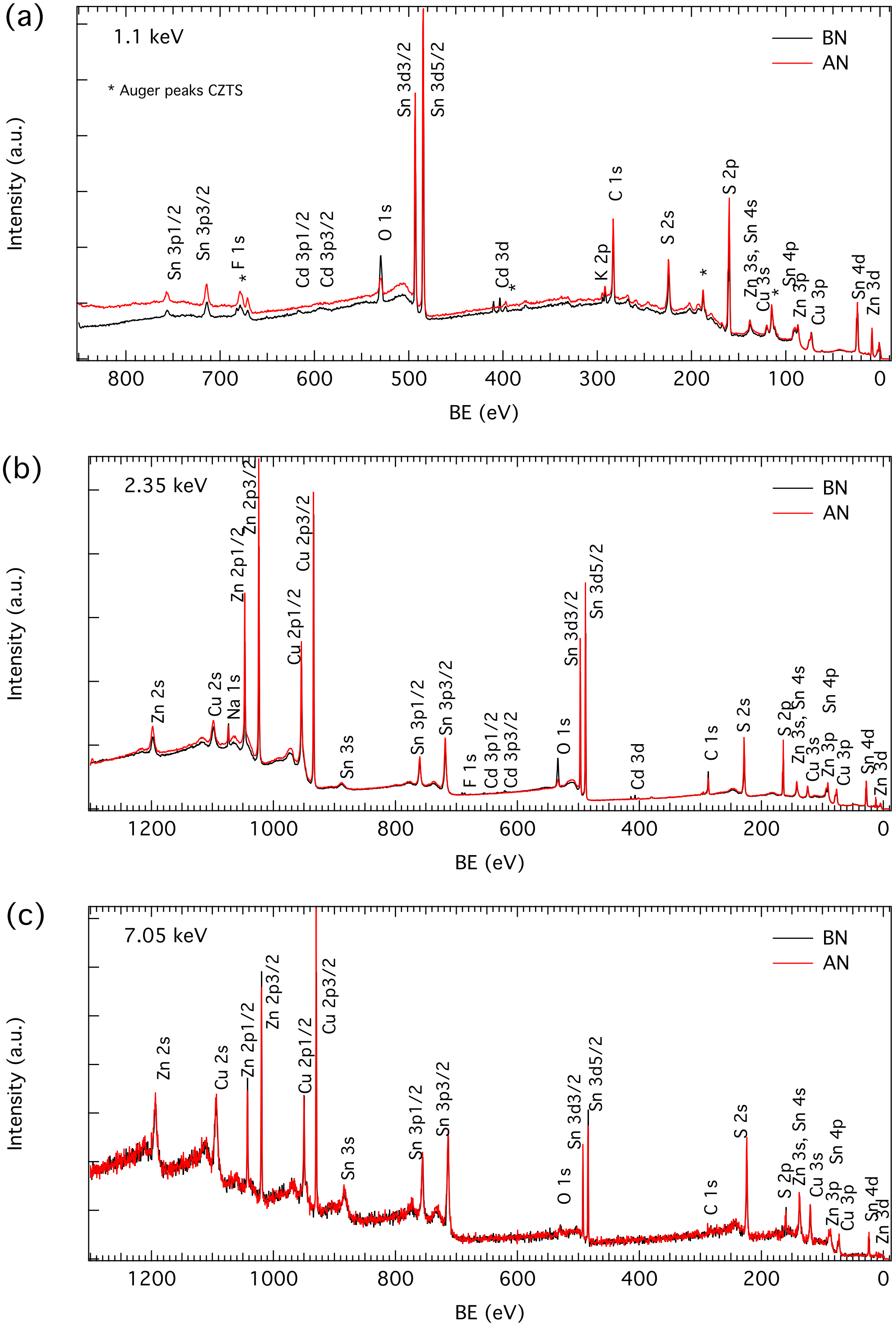

Figure S3: XPS and HAXPES survey spectra recorded at 1.1 (a), 2.35 (b) and 7.05 (c) keV for the CZTS samples before and after N_2 anneal. The most prominent lines are labeled. The following notation is used: BN = before N_2 anneal, AN = after N_2 anneal.

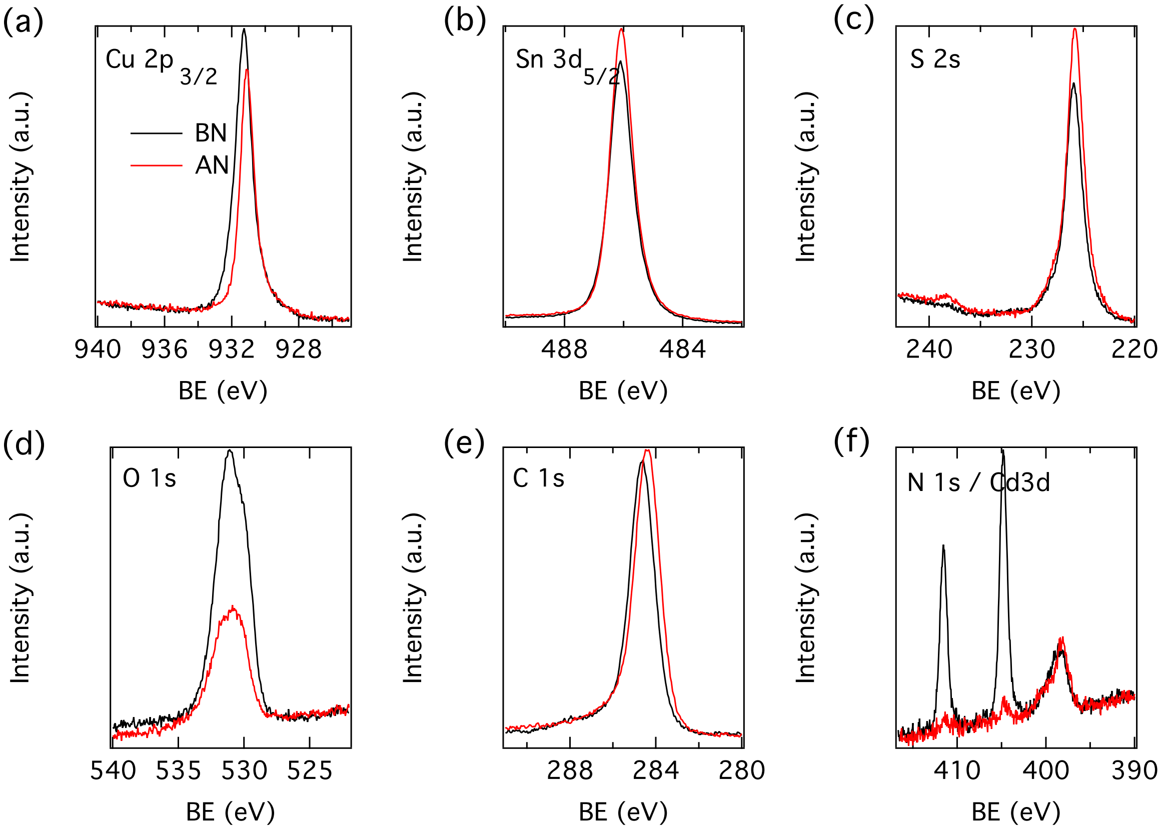

Figure S4: Photoemission spectra before and after N_2 anneal treatment of CZTS recorded with an excitation energy of 1.1 keV: (a) Cu $2p_{3/2}$, (b) Sn $3d_{5/2}$, (c) S 2s, (d) O 1s, (e) C 1s and (f) N 1s. The spectra have been aligned to Zn 3d peak (set to 10.0 eV). The following notation is used: BN = before N_2 anneal, AN = after N_2 anneal.

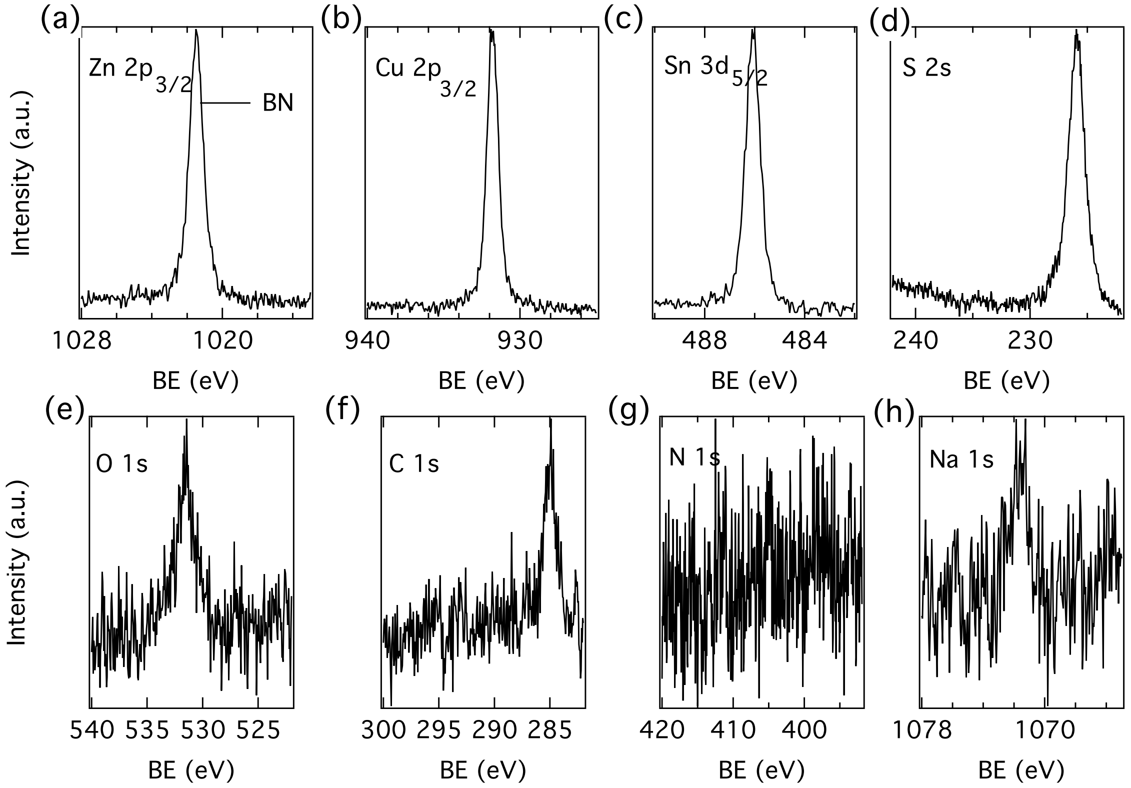

Figure S5: Photoemission spectra before and after N_2 anneal treatment of CZTS recorded with an excitation energy of 7.05 keV: (a) Zn $2p_{3/2}$, (b) Cu $2p_{3/2}$, (c) Sn $3d_{5/2}$, (d) S 2s, (e) O 1s, (f) C 1s, (g) N 1s and (h) Na 1s. The spectra have been aligned to Zn 3d peak (set to 10.0 eV). The following notation is used: BN = before N_2 anneal.

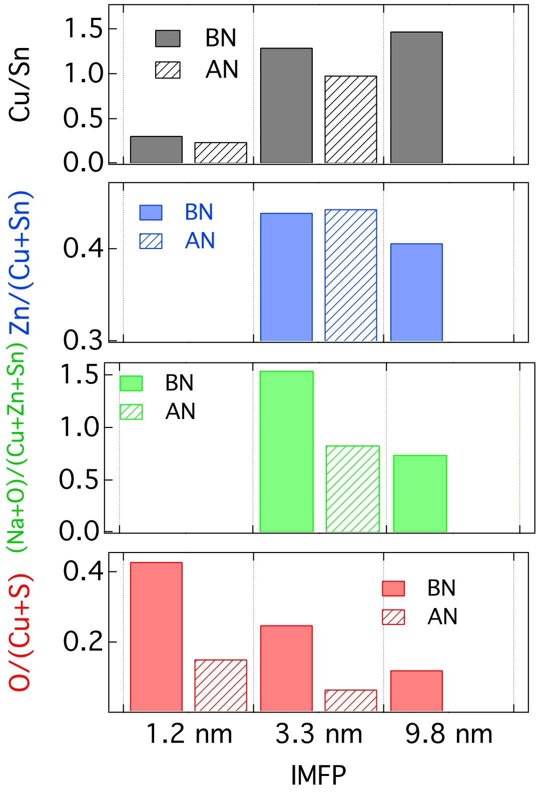

Figure S6: Relative composition analysis as calculated from the XPS/HAXPES data for both 1.1, 2.35 and 7 keV measurements. Note that Na 1s and Zn 2p measurements were not possible at the lowest photon energy employed (1.2 nm IMFP) and thus omitted in the composition analysis. The following notation is used: BN = before N_2 anneal, AN = after N_2 anneal.

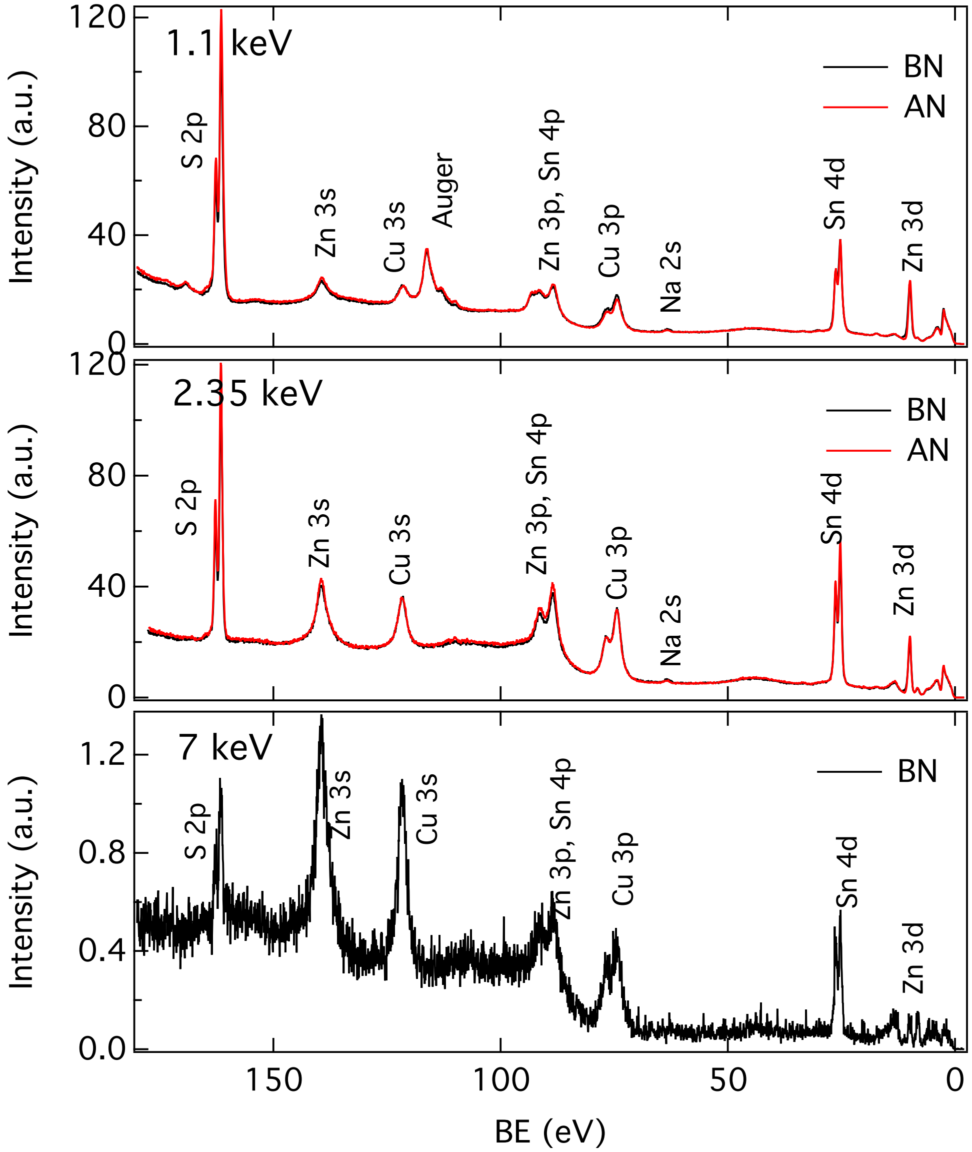

Figure S7: Extended valence band spectra before and after N2 anneal treatment of CZTS recorded with a photon energies of 1.1 keV (top), 2.35 keV (middle) and 7.05 keV (bottom), respectively. The main peaks are labeled and spectra have been aligned to Zn 3d peak (set to 10.0 eV). The following notation is used: BN = before N_2 anneal, AN = after N_2 anneal.

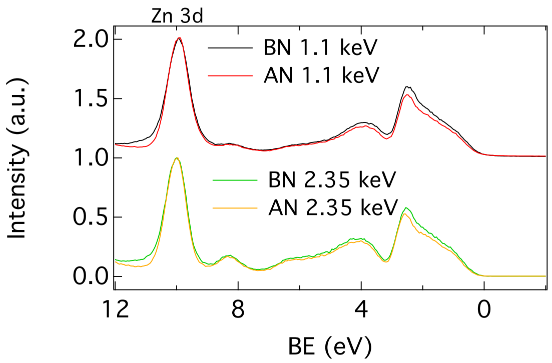

Figure S8: VB spectra before and after N_2 anneal treatment of CZTS recorded with a photon energy of 1.1 keV (top) and 2.35 keV (bottom), respectively. The spectra have been aligned to Zn 3d peak (set to 10.0 eV) as indicated. The following notation is used: BN = before N_2 anneal, AN = after N_2 anneal.

investigated. Thus, it has not been included in the compositional analysis.

Table S1 shows the change of the full width half maximum (FWHM) as a function of CZTS surface treatment and ZTO buffer layer deposition. An increase in the FWHM is observed for the Sn $3d_{5/2}$ and Zn $2p_{3/2}$ core levels for the ZTO/CZTS samples as compared to CZTS reference at both 3 and 9 keV, whereas the FWHM was similar for the Cu and S signals.

Table S1: FWHM [eV] of the Zn $2p_{3/2}$, Cu $2p_{3/2}$, Sn $3d_{5/2}$ and S 2s core levels for the investigated CZTS + ZTO samples as determined from the measurements performed at 3 keV and 9 keV, respectively. Data from CZTS reference is included for comparison.

Sample	Zn $2p_{3/2}$	Cu $2p_{3/2}$	Sn $3d_{5/2}$	S 2s
		3 keV		
CZTS	1	0.9	0.95	1.8
F	1.5	-	1.2	-
AE	1.5	-	1.2	-
AA	1.5	-	1.2	-
		9 keV		
CZTS	0.9	0.9	0.85	1.8
F	1.3	0.9	1.1	1.8
AE	1.3	0.9	1.1	1.8
AA	1.3	0.9	1.1	1.8

Figure S13 shows the attenuation behaviour of the different core levels from the CZTS + ZTO samples investigated recorded using an excitation energy of 9 keV. The peak areas were

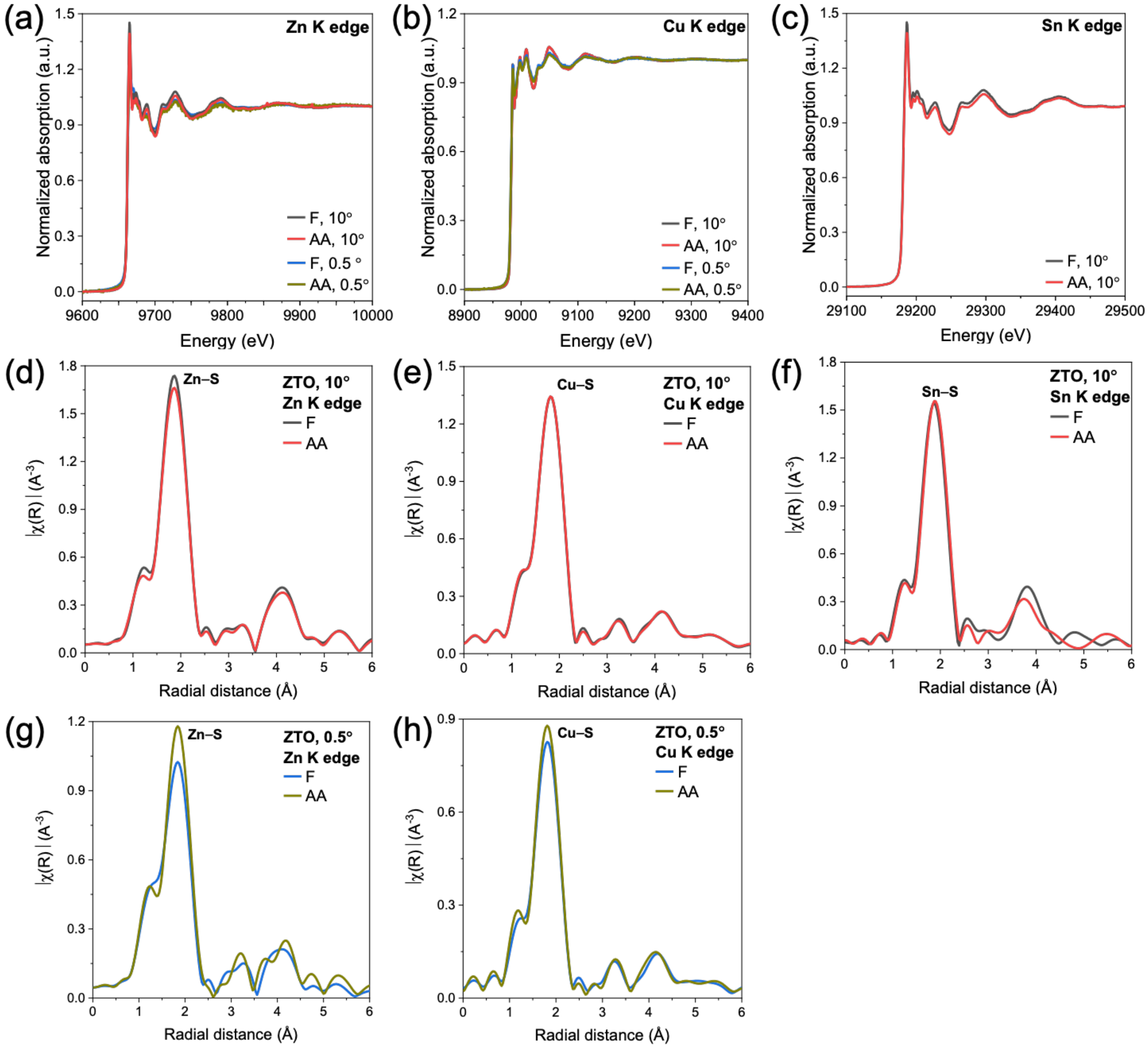

Figure S9: Raw XAS spectra of the Zn, Cu and Sn K edges recorded at 10 and 0.5 degrees incidence angles (a-c). EXAFS data (magnitude of the FT (k^2-weighted)) at the Zn, Cu and Sn K edges recorded for the fresh and air annealed samples at 10 degrees incidence angle (d-f) and 0.5 degree incidence angle (g-h). The Sn K-edge EXAFS at 0.5 degree is not included as the probing depth is much higher compared to the Zn and Cu K-edges and are thus not directly comparable.

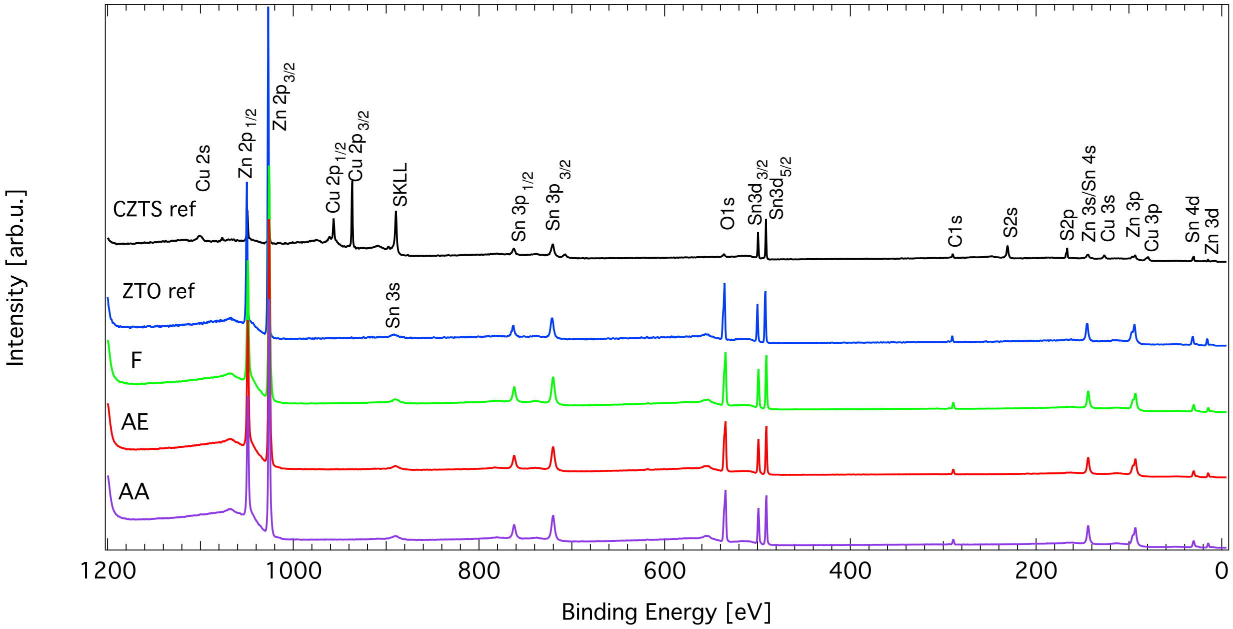

Figure S10: HAXPES survey spectra recorded at 3 keV for the ALD ZTO on non-treated (F) and surface treated CZTS samples (AE and AA) as well as reference samples (ZTO and CZTS ref) recorded with an excitation energy of 3 keV. The most prominent lines are labeled and the spectra have been vertically offset for clarity. The following notation is used: F=Fresh, AE=Air Exposed, AA=Air annealed.

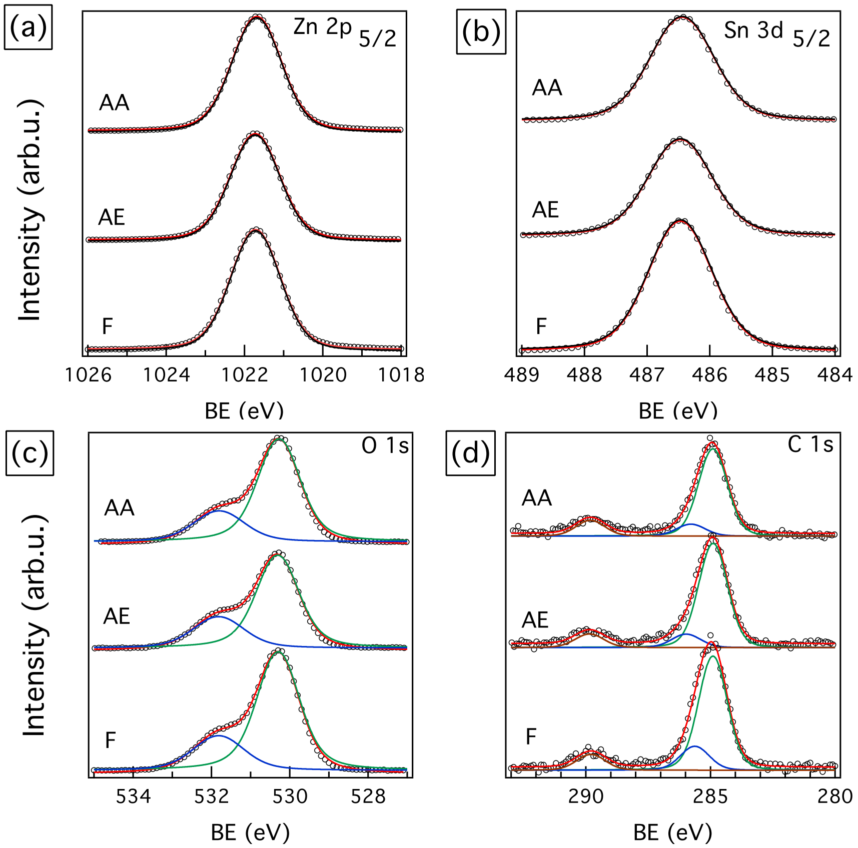

Figure S11: HAXPES data of ALD ZTO on F (bottom spectra), AE (middle spectra), and AA (top spectra) CZTS samples, recorded with an excitation energy of 3 keV: Zn $2p_{3/2}$ (a), Sn $3d_{5/2}$ (b), O 1s (c), C 1s (d). The spectra are displayed with the respective fits with Voigt profiles and a linear background subtracted. The following notation is used: F=Fresh, AE=Air Exposed, AA=Air annealed.

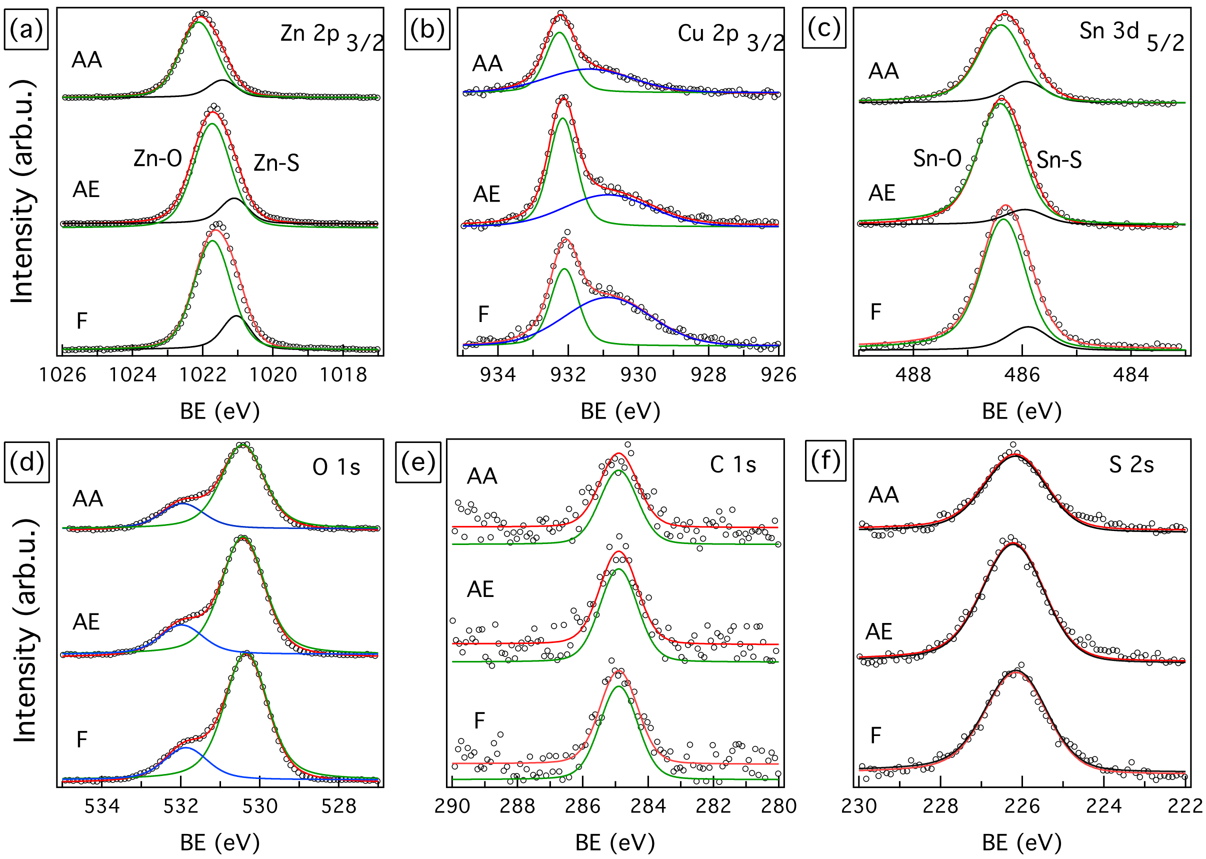

Figure S12: HAXPES data of ALD ZTO on F, AE, and AA CZTS samples, together with CZTS reference spectra recorded with an excitation energy of 9 keV: Zn $2p_{3/2}$ (a), Cu $2p_{3/2}$ (b), Sn $3d_{5/2}$ (c), O 1s (d), C 1s (e), S 2s (f). The spectra are displayed with the respective fits with Voigt profiles and a linear background subtracted. The following notation is used: F=Fresh, AE=Air Exposed, AA=Air annealed.

normalised such that the areas sum of all shown photoemission lines is 1 for each sample. The deconvoluted components of Sn and Zn core levels (i.e., Me-S and Me-O states) have been considered as indicated.

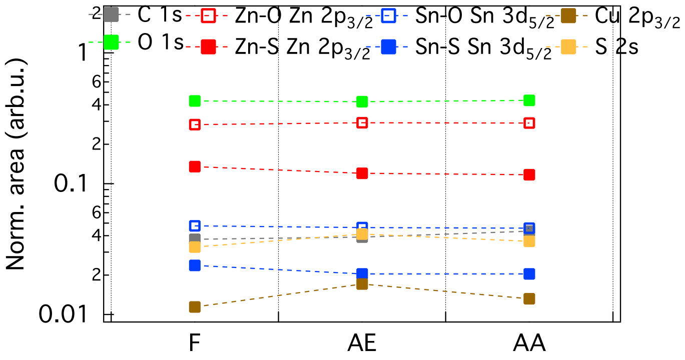

Figure S13: Evolution of the C 1s, O1s, Zn $2p_{3/2}$ (Zn-S and Zn-O components), Sn $3d_{5/2}$ (Sn-S and Sn-O components), S 2s and Cu $2p_{3/2}$ core level spectra for the investigated ZTO/CZTS samples exposed to different surface treatments at an excitation energy of 9 keV. The peak areas were normalised such that the areas sum of all shown photoemission lines is 1 for each sample. Note that a logarithmic y-scale has been employed to better visualise small contributions and dotted lines are only used to guide the eyes. The following notation is used: F=Fresh, AE=Air Exposed, AA=Air annealed.
